# Workshop on immunotherapy combinations. Society for immunotherapy of cancer annual meeting Bethesda, November 3, 2011

**DOI:** 10.1186/1479-5876-10-108

**Published:** 2012-05-28

**Authors:** Ivan Martinez Forero, Hideho Okada, Suzanne L Topalian, Thomas F Gajewski, Alan J Korman, Ignacio Melero

**Affiliations:** 1 Centro de Investigacion Medica Aplicada, Universidad de Navarra, Avda Pio XII, 55, 31008, Pamplona, Spain; 2 Department of Neurological Surgery, University of Pittsburgh School of Medicine, Pittsburgh, PA, USA; 3 Department of Surgery, Johns Hopkins University School of Medicine and Sidney Kimmel Comprehensive Cancer Center, Baltimore, MD, USA; 4 Biologics Discovery CaliforniaBristol-Myers Squibb, Milpitas, CA, USA; 5 Department of Pathology and Department of Medicine, University of Chicago, Chicago, IL, USA; 6 Clinica Universidad de Navarra, Pamplona, Spain

**Keywords:** Immunotherapy, Combination immunotherapy, Cancer vaccines, Chemotherapy, Anti-CTLA4, Anti-PD/PD-L1

## Abstract

Although recent FDA approvals on ipilimumab and sipuleucel-T represent major milestones, the ultimate success of immunotherapy approaches will likely benefit from appropriate combinations with other immunotherapeutic and/or non-immunotherapeutic approaches. However, implementation of ideal combinations in the clinic may still face formidable challenges in regulatory, drug-availability and intellectual property aspects. The 2011 SITC annual meeting hosted a workshop on combination immunotherapy to discuss: 1) the most promising combinations found in the laboratory; 2) early success of combination immunotherapy in clinical trials; 3) industry perspectives on combination approaches, and 4) relevant regulatory issues. The integrated theme was how to accelerate the implementation of efficacious combined immunotherapies for cancer patients. Rodent animal models are providing many examples of synergistic combinations that typically include more than two agents. However, mouse and human immunology differ in a significant number of mechanisms and hence we might be missing opportunities peculiar to humans. Nonetheless, incisive animal experimentation with deep mechanistic insight remains the best compass that we can use to guide our paths in combinatorial immunotherapy. Combination immunotherapy clinical trials are already in progress and preliminary results are extremely promising. As a key to translate promising combinations into clinic, real and “perceived” business and regulatory hurdles were debated. A formidable step forward would be to be able to test combinations of investigational agents prior to individual approval. Taking together the FDA and the industrial perspective on combinatorial immunotherapy, the audience was left with the clear message that this is by no means an impossible task. The general perception is that the road ahead of us is full of combination clinical trials which hopefully will bring clinical benefit to our cancer patients at a fast pace.

## The need for immunotherapy combinations

The long quest to attain clinical benefit in cancer patients by activating the immune system against such a deadly disease is finally paying off [1,2]. Exciting results have been recently reported with immunostimulatory monoclonal antibodies (mAbs) [3-6], with cancer vaccines [7] and with adoptive T cell therapies [8,9]. Success and optimistic perspectives have brought steadily increasing industrial interest in this area of research. At this point in time the private and public sectors are moving the field of immunotherapy forward at an unprecedented pace.

An important lesson to be learned from past major victories against human diseases is that combined treatments are often the key to synergistically achieve clinical success. Let us consider for instance antibiotic combinations for Mycobacterium tuberculosis [10], combined chemotherapies for pediatric acute lymphoid leukemias [11] or HAART for AIDS patients [12]. It is unlikely that individual immunotherapeutic agents, even considering the most efficacious examples of them, will be ultimately successful as monotherapy. In fact abundant data in mouse models provide very solid evidence for examples of treatment combinations acting synergistically [13-16]. Such preclinical examples of synergistic combinations include combinations of multiple immunotherapeutic agents and combinations of immunotherapies with other modalities of cancer treatment. These combinations will be required because of multiple resistance mechanisms that tumors utilize to evade immune responses as well as the numerous mechanisms that normally limit host immune responses in health and disease. The wealth of knowledge about tumor evasion of immune rejection together with preclinical testing should hasten a number of initiatives to explore combinations in the clinical arena.

The following four themes were selected for the focus of a workshop at the 2011 SITC meeting devoted to immunotherapy using combinatorial treatment approaches: (i) How to best identify at an early stage of development the most efficacious combinations; (ii) How to recognize problems of safety early in development; (iii) How to approach combinatorial treatments from a regulatory point of view; and (iv) How to deal with legitimate interests of intellectual/industrial property, commercialization and risk-taking in the life-cycle of innovative drugs. Authoritative speakers addressed these points and the sessions were designed with ample discussion time to facilitate dialogue among participants with a diverse range of expertise and experience.

## Palettes of immunotherapy agents to be combined

**Figure 1:**
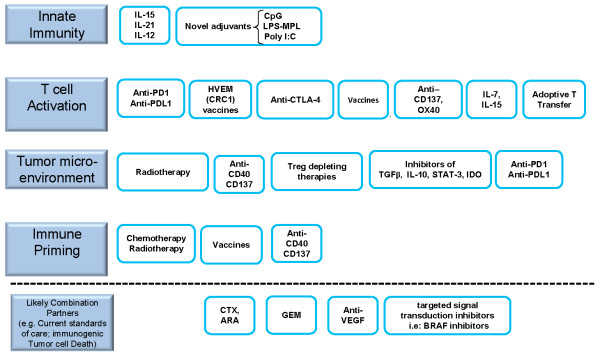
**SITC meeting overview of cancer immunotherapy and potential combination strategies within four key categories: Innate immunity, T cell activation, Tumor micro-environment, immune priming and boosting.** Treatments are grouped in categories according to mechanism of action. Some of the listed products intervene in more than one category. A rational for combinations with elements taken from each of the different mechanistic categories should lead to optimized efficacy, while maybe minimizing autoimmune adverse effects. (CTX = cyclophosphamide, ARA = arabinoside, GEM = gemcitabine).

As a result of decades of investigation we have acquired clinical evidence for the therapeutic activity of a number of immunotherapy agents that now become the focus of potential combinatorial therapies. The palette of immunotherapies includes enhancers of dendritic cell function, vaccines, adoptive T cell transfer including genetically modified T cells, immune checkpoint inhibitors and agents to neutralize or inhibit suppressive cells, and cytokines. The therapeutic immune response against tumors involves different layers that are to be tampered with in order to orchestrate a clinically meaningful outcome. As shown in Figure 1, these include factors modulating innate immunity, modulation of T cell activation and inhibition, factors modifying the tumor microenvironment, and means to provide or attain antigen priming or boosting. The abundance of promising products raises the following crucial question: Which agents warrant focused funding for clinical development? [17] Their potential for rational combinations among them should be a major guidance factor for selection.

Kim Margolin (University of Washington) reported the coordinated efforts of the Cancer Immunotherapy Trials Network (CITN) to build upon the collective experience and accumulated wisdom of “the field” to prioritize drugs, regimens and most importantly to conduct combination trials with high priority agents. In a series of workshops promoted by the NCI with broad scientific input, IL-15, anti-CD40, anti-PD-1, and IL-7 were defined as the most compelling for providing patient benefit in the near term. Of note, anti-CTLA-4 mAbs were excluded from discussion since ipilimumab had already been submitted for FDA approval.

IL-15 is a cytokine that increases CD8 and NK cells both in numbers and activity [18]. A phase I clinical trial using recombinant IL-15 includes an escalation dose of rhIL15 (0.50, 1.0, 2.0 and 3.0 mcg/kg/day) in patients with melanoma, renal and colon cancer (NCT01021059). The primary aim is to select outpatient doses and schedules to safely enhance various T-cell (CAR, vaccines) and NK cell (ADCC) therapies An extensive immune monitoring protocol will also be performed. Preliminary results presented at the SITC meeting from the first-in-human clinical trial (Tom Waldmann, NCI) suggest that humans are more sensitive than primates to intravenous recombinant IL-15 and at least in bolus infusion there were unexpected signs of acute toxicity at low doses including severe hypotension and high fever. Continuous infusion may be a way to overcome this problem, and subcutaneous administration is being considered.

IL-7 is a homeostatic growth factor for T cells. IL-7 is well tolerated in humans in a broad dose range (3–60 μg/kg/dose) and has been tested in patients with advanced cancers [19]. It increases the number of circulating CD4 and CD8 T cells and promotes spleen and lymph node enlargement [20]. IL-7 preferentially expands naïve T cells while reducing Treg numbers and function. CITN is proposing three clinical trials combining IL-7 with vaccines in prostate cancer (Sipuleucel T-Dendreon), melanoma (MAGE3 protein - GSK) and chimeric antigen receptor transfected T cells in ovarian cancer (CARs).

## Vaccines

Malignant tumors display a multitude of genetic abnormalities that can generate tumor specific antigens (TSAs) recognizable by the immune system. Dendritic cells can take-up TSAs and depending on the environmental conditions, present the antigens in the tumor microenvironment or at lymphoid organs to mount, sustain or abrogate the antitumor immune response. Cancer vaccines try to exploit these mechanisms by using potent antigens combined with the appropriate danger signals to initiate and maintain an antitumoral response. One of the most extensively studied tumor antigens is the tumor specific idiotype present in B cell lymphomas. Larry Kwak (M.D. Anderson Cancer Center) discussed his experience with anti-idiotype cancer vaccines in follicular lymphoma. The tested products are composed of tumor-derived Ig-containing tumor specific idiotypic determinants. The malignant cell-derived Ig is covalently linked to KLH, which serves as a carrier protein and an immune stimulant. The vaccine is injected with GM-CSF used as an adjuvant to attract and differentiate dendritic cells at the site of subcutaneous vaccination. In a Phase II clinical trial the majority of patients mounted specific CD8+ T cell responses against the idiotype [21]. About half of the subjects are still in remission (>10 years) and never have received Rituximab. These positive results paved the way for the design of a Phase III clinical trial. Patients with follicular lymphoma were randomized after complete remission to receive the full vaccine or the components of the vaccine without the specific Ig. In a modified intent to treat analysis the vaccine prolonged disease-free survival. A subset analysis revealed that IgM isotype lymphomas have a better response to the vaccine when compared to lymphomas of IgG isotype [22].

When these idiotype vaccine trials were started, rituximab (anti-CD20, now a standard of care in lymphoma) had not been approved yet. In a trial involving mantle cell lymphoma patients, vaccination was administered after remission induction with six cycles of EPOCH chemotherapy plus rituximab. Median overall survival of 8.7 years compared very favorably with historical controls in which the expected survival is about 3–4 years. This is important because depletion of normal B by the anti-CD20 mAb cells does not seem to counteract the benefit of vaccination [23].

Hormone refractory prostate cancer is a very suitable therapeutic target for development of a cancer vaccine approach. In this disease there is a long interval from primary diagnosis to metastatic disease and serum PSA (doubling time velocity) can be used as a surrogate marker for therapeutic response or disease recurrence. Recently, Sipuleucel-T (a product containing PBMC pulsed with a fusion protein of prostatic acid phosphatase (PAP) with GMSF) has been approved as a first line treatment in castrate resistant metastatic prostate cancer. This autologous cellular product, derived from sequential leukaphereses, is complex and may combine the effects of vaccinating against PAP and adoptive transfer of primed T cells in subsequent infusions [24]. Additional prostate cancer vaccine strategies with the injection of viral vectors are currently in clinical trials. Philip Arlen (National Cancer Institute) described the experience with Prostvac, a viral vector encoding transgenes for PSA as a tissue restricted antigen, and three immune costimulatory molecules (B7.1, ICAM-1, and LFA-3). A Phase II clinical trial reported an 8.5-month improvement of overall survival in patients with prostate cancer [25]. Combinations of vaccines with chemotherapy, radiotherapy and hormone blocking therapy are expected. A randomized trial is ongoing for patients with nonmetastatic CRPC receiving vaccine (rV-PSA/B7.1, rF-PSA) or androgen receptor antagonist (Nilutamide) with crossover at progression. It is very enticing to observe that there was a 59% survival in patients receiving the vaccine first compared to 39% of those patients that receive nilutamide before progression [26]. Vaccination requires in some instances a long period of time to observe clinical benefit and for this reason it seems not advisable not to use vaccines after progression in CRPC.

## Monoclonal antibodies modulating the immune system

T cells require different signals for appropriate expansion and acquisition tumoricidal functions [27]. Signal 1 is initiated after cognate T-cell receptor binding to a MHC-peptide complex on the surface of an antigen-presenting cell. Co-regulatory molecules including CD28 and CTLA-4 that bind to CD80 and CD86 deliver or modulate signal 2. Moreover, other co-regulatory interactions and paracrine cytokines, both stimulatory and inhibitory, shape and direct the type of response (signal 3). Crucially, negative regulators modulate the grade of T cell activation and protect the organism from deleterious effects mediated by uncontrolled T cell activation. CTLA-4 is one of these co-inihibitory molecules expressed on activated T cells. It binds CD80 and CD86 with an affinity at least 100-fold higher than CD28. A fully human mAb blocking this interaction has been approved for the treatment of metastatic melanoma [5,6].

PD-1 is a co-inhibitory receptor that shows homology with CD28 and whose expression is induced following activation on CD4+ and CD8+ T cells as well as on other immune cells. Its cognate ligands are the members of the B7 family PD-L1 (B7-H1) and PD-L2 (B7-DC). PD-1 triggering on the T cell leads to a non pro-apoptotic inhibition of T cell proliferation and activation dependent on recruitment of a tyrosine phosphatase that represses signaling at immune synapses [28]. In animal models of infectious disease, PD-1 is upregulated in both acute and chronic LCMV infection but stays high in chronic infection marking exhausted T cells. Indeed, PD-1 is considered as a decisive exhaustion marker along with LAG-3, CD160, BTLA, and 2B4. Three antibodies against PD-1 have started clinical trials in patients with advanced cancers. Early reports suggest excellent clinical activity with conspicuously long-lasting responses and disease stabilization [2,29]. In addition there is interest in selectively modulating PDL1 given other inhibitory interactions of this molecule [30] and the anti-apoptotic signals of PDL1 to tumor cells [31].

HVEM is a member of the TNFR superfamily with multiple ligands, both stimulatory and inhibitory. Gordon Freeman’s group (Dana Farber Cancer Institute) has demonstrated that deletion of HVEM cysteine rich domain 1 (CRD1) interferes with the binding capacity of the inhibitory receptors BTLA and CD160. In contrast, HVEM lacking CRD1 is a stimulatory receptor through interactions with LIGHT. B16 melanoma expressing HΔD1 (HVEM with deletion of CRD1 domain) becomes immunogenic and mice harboring irradiated B16-HΔD1 survive longer compared to B16 recipient mice. Treatment efficacy is further improved with the concomitant use of anti-CTLA4 and anti-PD-1 mAbs. The combined strategy greatly enhances lymphocyte trafficking into tumor sites. Optimal immunotherapy may involve a combination of blockade of inhibitory signals and provision of costimulatory signals as if releasing the brakes and stepping on the gas pedal of a car [3]. The above strategy may be used to endow cellular vaccines with immune stimulating properties, through genetically introduced expression of molecules such as GM-CSF, Flt-3-ligand, ICOS-ligand and others [32,33].

CD40 is a costimulatory molecule expressed on antigen presenting cells and frequently on neoplastic cells with no intrinsic kinase activity. CD40-CD40L interactions facilitate dendritic cells (DCs) to increase expression of CD80 and CD86 and threby activate T cells. A fully human mAb (CP-870,893) that recognizes CD40 has been developed and shown to activate human DCs. This mAb is currently in clinical trials for several types of malignant tumors. Robert Vonderheide (University of Pennsylvania) described his experience with anti-CD40 mAb in pancreatic cancer [34]. Agonist CD40 mAb combined with gemcitabine induced major clinical remissions in patients with pancreatic adenocarcinoma. CD40-dependent treatment effects can be reproduced in a genetically engineered mouse model of the disease. Strikingly, the therapeutic mechanism fol-lowing CD40 activation is not T cell-dependent, but rather is macrophage-dependent. Intriguingly, CD40-activated macrophages rapidly infiltrate and kill tumor cells, and facilitate depletion of tumor stroma. CD40 can work effectively to induce tumor infiltrating T cells but requires gemcitabine for antigen priming outside the tumor microenvironment.

Monoclonal antibodies that bind stimulatory or inhibitory receptors are excellent pharmacological tools to magnify the role of receptor-ligand pairs in cancer immunotherapy. However, there are some unknowns about mechanisms underlying the action of these mAbs. Martin Glennie (University of Southampton) discussed the physiology of Fc receptors and effects on the function of anti-CD40 mAb [35]. This British group concomitantly with Li and Ravetch [36] at Rockefeller University (NYC) found that the isotype of the mAb plays a crucial role in the activity of anti-CD40 mAb. IgG1 is optimal in mouse models due to its ability to bind FcγRIIB in vivo, thereby allowing cross-linking of the FcR receptor upon cell-to-cell contact with the responding leukocytes. Early results from the same group suggest that other immunostimulatory mAbs are FcR cross-linking dependent. Understanding these mechanisms will allow fine-tuning of agonistic mAbs and may optimize the benefit:toxicity ratio of these agents.

The immune system recognizes CpG oligonucleotides through TLR9 receptors that activate B-lymphocytes and DCs. Intratumoral injection of CpG after radiation therapy promotes systemic lymphoma eradication in mice [37] with interesting results in humans [38]. CpG in situ vaccination induces anti-tumor T cells that express CD45RO and CD137. This type of treatment is practical (no need for a customized product), safe and repeatable. The group of Ron Levy (Stanford University) identified combinations with immunostimulatory mAbs that extend the efficacy of CpG intratumoral injections in murine models. In transplanted tumors, OX40 and CTLA4 were highly expressed on tumor-infiltrating lymphocytes compared to T cells in the spleen or draining lymph nodes. Most OX40/CTLA4+ T cells infiltrating tumors were FOXP3+ regulatory T cells. When mice were intratumorally treated with anti-OX40 and anti-CTLA4 mAbs, FOXP3 + CD4+ T cells were depleted and established tumors eliminated using as low as 1/100 of the needed dose when given systemically. This intratumoral route was also capable of inducing the rejection CNS leukemia metastases. The message is: “treat locally, cure systemically”.

## Opportunities for synergy with non-immune therapies

Vemurafenib is a BRAF inhibitor that improves overall and progression-free survival in metastatic melanoma patients. Vemurafenib selectively blocks the aberrant serine/threonine kinase activity arising from the common BRAF V600E mutation in melanoma cells. The good news is that this drug does not eliminate immune cells and does not interfere with MAPK signaling in T lymphocytes. In fact, vemurafenib has been reported as an immunosensitizer because it increases the availability of tumor antigens and seems to stimulate NK activating receptors. Synergy with immunotherapy is at least theoretically expected. The group of Antoni Ribas (University of California Los Angeles) has generated a murine melanoma line, SM1, harboring BRAF V600E. SM1 cells are moderately sensitive to vemurafenib and were transfected with chicken ovalbumin as a model antigen. BRAF inhibitors do not change gp100 melanoma antigen expression in SM1 cells. In combination therapy experiments with adoptive T cell transfer (OT-1 T cells or Pmel TCR-transgenic lymphocytes) vemurafenib is highly efficacious in mice bearing these tumors. This compound does not increase T cell homing to tumors but enhances cytokine production by tumor infiltrating lymphocytes. A phase I/II clinical trial combining vemurafenib and ipilimumab in metastatic melanoma is currently recruiting patients (NCT01400451).

Chemotherapy is one of the pillars of conventional cancer treatment. During the last few years the group of Laurence Zitvogel and Guido Kroemer in Paris has postulated and defined a key role for the immune system in the mechanisms of action of chemotherapy. In particular some drugs such as anthracyclines and oxaliplatin require a functional immune system to exert therapeutic effects [39]. Immunogenic cell death is accomplished by a complex molecular machinery that ultimately exposes calreticulin at the cell surface of cancer cells, as well as ATP and HMGB1 release. In a recent series of experiments using tumor cells deficient for ATG5 or ATG7, Zitvogel et al demonstrated that autophagy is essential for immunogenic cell death [40].

As an important corollary for those chemotherapeutic agents that do not induce immunogenic cell death, an additional drug can take over the activation of the missing hallmark of immunogenic cell death. An extensive screening by Zitvogel’s group has discovered a previously undescribed role for digoxin in cancer. Cardiac glycosides in mice promote immunogenic cell death when combined with non-immunogenic chemotherapeutic drugs such as mitomycin-C. Interestingly, in a retrospective analysis, cancer patients receiving concomitantly chemotherapy and digoxin exhibited improved overall survival when compared to patients who were not on digoxin. This effect was restricted to select tumor types including breast cancer, colorectal cancer, hepatocellular carcinoma, head and neck cancer but not prostate or lung cancer (Zitvogel et al unpublished data).

Malignant cells require new blood vessels to grow. The major proangiogenic factor VEGF can also contribute to the immunosuppressive environment found in cancer tissues [41]. Monoclonal antibodies that inhibit VEGF (i.e. bevacizumab) are used to treat cancer patients and can enhance the number of activated DCs and impair the function of regulatory T cells [42].

During the clinical testing of ipilimumab, Stephen Hodi (Dana Farber Cancer Institute) observed that this anti-CTLA-4 mAb induced hemorrhagic necrosis and immune mediated vasculopathy [43]. Ipilimumab also promotes the development of antibodies to VEGF-A in long-term surviving melanoma patients [44]. In addition VEGF may drive immunosuppressive loops. Therefore, anti-CTLA4 mAb might synergize with anti-VEGF antibodies. To test this hypothesis, Dr. Hodi designed a Phase I clinical trial for the combination of ipilimumab and bevacizumab in melanoma patients. Post-treatment biopsies demonstrated morphologic changes in tumor blood vessels and extensive immune effector cell trafficking. In peripheral blood a consistent increase in both CD4 and CD8 memory T cells was observed. This combination trial is still in progress (NCT00790010) with promising results in terms of clinical activity and safety in melanoma patients. At the end of his talk, Dr. Hodi commented on his three-year ordeal to (i) secure cooperation between the two companies owning the drugs, (ii) obtaining funds for the trial and (iii) the process of getting the regulatory approval of the trial by the FDA.

## Surmounting business and legal obstacles for cooperative development of combination therapies

Combination immunotherapy in cancer often requires navigating through multiple layers of regulatory and business challenges. Raj K. Puri (Division of Cellular and Gene Therapies, Office of Cellular, Tissue, and Gene Therapies/CBER/FDA) discussed clear definitions and information resources about combination products and combinatorial therapies. A combination is composed of two or more therapeutic components (drug, device, or biological product) required for an intended effect (i.e. mAb combined with a therapeutic drug). A combinatorial product is a therapy combining multiple components. The revision process for safety and efficacy of a combination product is assigned to a FDA division according to the primary mode of action (CBER for biologics, CDER for drugs, CDRH for device) with consultancy from the Office of Combination Product. The FDA provides online resources for clarification (http://www.fda.gov/BiologicsBloodVaccines/NewsEvents/ucm232821.htm).

There are important ongoing efforts to coordinate IND management without revealing trade secrets, and to coordinate the FDA with regulatory organizations in Europe and Asia. These steps should simplify the required evaluation processes and focus on safety.

Pharmaceutical companies could be reluctant to participate in combination trials when their agent is still under development as a monotherapy. David Bonk (Bristol Myers Squibb) described legal and logistical issues in the clinical evaluation of cancer immunotherapy combinations. Legal barriers come in different flavors (intellectual property, contracts, antitrust law, product liability) but in actuality, he argued, there are very few true legal obstacles. However, as expressed by Dr Bonk “there are transactional dynamics that are sometimes expressed in legal terms”. In his experience the most common themes are failure to grasp the key interests and concerns of the other negotiating partner and the desire of one party to extract more value from the partnership than is warranted by its own contribution or assets. It is important to keep in mind the patients that could benefit for the use of the combination.

## Conclusions

Comments from audience comments reflected the excitement of the different stake holders. Importantly, senior management at leading pharmaceutical companies is increasingly convinced of the translational value of these therapeutic combinations. The many difficulties are clearly perceived but there is also optimism in terms of foreseeing unprecedented efficacy.

We are at the dawn of a new and exciting era: the art and science of combinatorial immunotherapy will keep many of us busy for years to come. This is no longer a quixotic task, but a time of opportunity. Clinical translation in this new front in the war against cancer should be given the highest priority and facilitated by regulatory bodies. Drug companies, basic scientists and clinical investigators are coming together. An army of visionaries will be needed to succeed in the war against cancer. SITC will provide the environment to assemble such an army into the future.

## Competing interest

IMF: None to declare.

HO: None to declare.

ST is an uncompensated consultant for Bristol-Myers Squibb, and receives sponsored research support from Bristol-Myers Squibb. Her spouse is a consultant for Amplimmune Inc.TG Has served as a consultant for Bristol-Myers Squibb, Merck, Roche/Genentech, Eisai, Incyte, and GSK-Bio.

TG Has served as a consultant for BMS, Merck, Roche/Genentech, Eisai, Incyte, and GSK-Bio.

AK: is a full time employee in Bristol-Myers Squibb.

IM Is a consultant for Bristol Myers Squibb, Miltenyi biotec and Pfizer.

## Authors’ contributions

IMF and IM prepared the first draft of the manuscript. AK provided Figure 1. HO, ST, TG and AK revised the manuscript. All authors read and approved the final manuscript.
